# The Vision of a Green(er) Scientific Conference

**DOI:** 10.1289/ehp.1307302

**Published:** 2013-08-01

**Authors:** Nino Künzli, Martina S. Ragettli, Martin Röösli

**Affiliations:** Swiss Tropical and Public Health Institute, Basel, Switzerland and University of Basel, Basel, Switzerland, E-mail: Nino.Kuenzli@unibas.ch

The success of *Environmental Health Perspectives* is connected to the careers of many members of the International Society of Environmental Epidemiology (ISEE), the International Society of Exposure Sciences (ISES), and the International Society of Indoor Air Quality and Climate (ISIAQ)—three societies that will hold, for the first time ever, a joint annual meeting in 2013. The conference, “Environment and Health—Bridging South, North, East and West” (http://www.ehbasel13.org/), will be hosted by the Swiss Tropical and Public Health Institute in Basel, Switzerland. Basel, located at the intersection of three countries on the borders of the Rhine, became a hot spot of research in environmental health after a fire at a nearby chemical plant in 1986 (Ackermann-Liebrich et al. 1992; Capel and Giger 1988). Only 6 months after the Chernobyl disaster, Basel’s citizens woke up not only to a toxic smell but also to a river suddenly turned red by the release of agrochemicals into the Rhine, a result of runoff from the tons of water used by the firefighters. The event happened approximately 10 km upstream from Basel and led to the massive destruction of wildlife in the Rhine.

**Figure 1:**
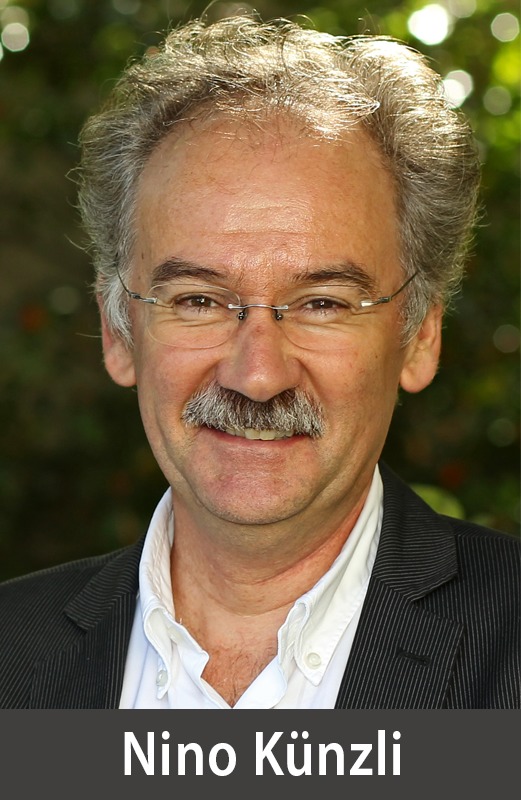
Environmental and economic trends in Switzerland, 1990–2010. Abbreviations: GDP, gross domestic product (in 1,000 million Swiss francs); Vkm, vehicle kilometers driven per year (in 1,000 millions); PM_10_, ambient air concentration of particulate matter ≤ 10 μm in diameter (annual mean in μg/m^3^); NO_x_, annual emissions of nitrogen oxides (in 1,000 tons).

The accident gave rise to a strong “green movement” that influenced scientists, policy makers, industry, and society. The event gave a further boost to successful and, at that time, rather unique international efforts to protect the Rhine ecosystem through treaties and alert systems (Dieperink 1998). The birth of the ISEE 25 years ago was enthusiastically welcomed by Basel researchers who shared the vision that improvements can occur only through solid research that provides the basis for evidence-based policies. The first ISEE meeting gathered some 100 scientists from just a handful of countries, including one Basel delegate. What followed was a strong Swiss research focus on air pollution, its health effects, and its public health impact (Braun-Fahrländer et al. 1992; Downs et al. 2007; Künzli et al. 2000; Zell et al. 2010). The emerging evidence of adverse health effects became the underlying theme of sustainable clean air policies (Künzli and Perez 2009). As a consequence, air quality continuously improved despite continued economic growth and associated expansion of vehicle use (Figure 1). Air quality improvements yielded relevant health benefits, both in children (Bayer-Oglesby 2005) and adults (Downs et al. 2007; Künzli et al. 2009; Schindler et al. 2009).

Today, the Rhine is considered to be cleaner than ever and has become a prime swimming resort for its citizens. In 2013, Basel will host this meeting (http://www.ehbasel13.org/); approximately 1,500 scientists from > 70 countries will discuss the latest in environmental and health research and report the evidence for policies that protect and foster the health of people and the ecosystem.

As scientists, we notice the environmental and climate footprint of international meetings. Thus, as conference organizers, we considered the environmental footprint of our own annual conference and how best to minimize it. Most scientists recognize that no matter what technologies we have or will see in the future, nothing can replace the added value and benefits for research and scientific networking of in-person get-togethers. Once in a while, researchers must see each other to exchange, interact, question, debate, or simply chat and laugh about their work and ideas. Thus, inevitably, scientific societies continue to have in-person meetings such as the one in Basel. The question is not whether to have or not have meetings, but how to run a “green” or “sustainable” international conference.

From the very beginning, the local organizing committee for the Basel conference had committees for the organization and scientific content of the meeting, as well as one for addressing environmental issues. The environment committee built on experiences and strategies from previous conferences, such as ISEE 2011 and 2012, and considered event guidelines, including information from the University of Basel (Lötscher et al. 2013) and the Swiss Federal Institute of Technology Zurich (Brunner and Elmer 2009). Obvious targets include the limitation of print material (with a printed program only upon request); the choice of regional, seasonal, and organically grown food; and reduced meat consumption. Indeed, the four lunch buffets will be vegetarian.

As simple as it sounds, it takes a lot of effort to implement such strategies because they are not yet standard (the “default”). Setting and promoting these new environmental strategies require multiple exchanges with all partners. For example, we asked caterers that all food served at the conference be seasonal, organic, and grown locally where possible (there are many organic producers in the area near Basel) and that those products not grown in Europe be traded fairly. How can it be that such requests remain “exotic,” while the default lunch buffet in Basel would include mangos shipped from Mexico or Brazil (organically grown upon request) and a glass of wine from Chile or Australia, which is offered at a lower price than wines from the Basel region? Why does the request to serve fair-trade coffee require the emptying of coffee machines because machines are “usually run with normal coffee”? What makes organic coffee so abnormal if it is produced and traded fairly and provides the growers a decent living without putting their health at risk from the use of pesticides (Maroni et al. 2006)?

Taken together, these footprints are very small compared with the greenhouse gases produced by participants traveling to Basel (Table 1). Based on the origins of the 1,400 current early registrations, attendees will travel a total of approximately 14 million km. Instead of asking participants to voluntarily compensate their CO_2_ (carbon dioxide) footprints (> 1.5 tons per average participant), we decided to run a CO_2_ neutral conference. By contracting South Pole Carbon (http://www.southpolecarbon.com), a firm committed to fighting climate change, the Basel conference committee chose two CO_2_ compensation projects—one in Uganda (efficient cooking stoves) and one in China (energy from waste gas)—that aim to reduce CO_2_ emissions and to improve the quality of life of the local population (ehbasel13.org 2013).

**Table 1:** Carbon footprint of Basel conference participants.

Characteristic	Region/travel description	Estimate
Geographical region of origin (%)	Europe	41
North America	28
Asia and Australasia	26
South America	3
Africa	2
Sum of travel distance (in 1,000 km)	Europe	1,080
Non-Europe	13,210
Travel mode of participants (%)	Airplane	81
Train	12
Other	7
CO_2_ footprint total (tons)^*a*^		2,202
Origin of footprint		
Air travel (%)	From/within Europe	8
From non-European countries	88
Other types of travel (%)		2
Venue, catering, print material (%)		2
Estimates are based on the origin of the first 1,400 registrations (1June 2013) and are expected to reflect approximately 90% of the final number of participants. ^***a***^Calculations done by South Pole Carbon (http://www.southpolecarbon.com/).		

Compensation for the inevitable CO_2_ footprint of the entire conference (Table 1) corresponds to approximately 20 Swiss francs per participant. We deliberately avoided itemizing these costs into the registration fees because the costs of the venue (~ 320 Swiss francs per participant), the food and coffee (a similar amount), and submission and review of abstracts are never itemized. CO_2_ compensation should also be part of these standard costs. Of course, the prime objective must be reduction rather than mere compensation of the footprint. In this context, compensation does not buy indulgences, but rather it results in an actual mass balance for CO_2_ by means of projects that otherwise are unlikely to be realized.

We realize that there is still a long way to go to accomplish the “green” goals of a conference with the same certainty and standards used to pursue scientific and organizational targets. In the planning process, scientific uncertainties or lack of evidence created challenges for making the best environmentally sound decisions within a limited time frame. The “green club” of the local organizing committee researched many issues, but not all resulted in clear-cut guidance. For example, there was no final conclusion on the most sustainable solution for distributing drinking water in refillable bottles or cups. The committee explored several options for containers [PET (polyethylene terephthalate), bioplastics, aluminium, stainless steel, and glass], but evaluating all the materials with regard to recyclability, potential re-use after the conference, production, costs, and health aspects of the materials is complex. Most important, organizing conferences in a greener way could require changes in standard practices and resource allocations that may frustrate conference organizers, if not participants. For example, the society of scientists has not yet reached the stage where a printed program booklet could be entirely replaced by an electronic version. During the registration process, participants were asked to give their preference, and some two-thirds opted for electronic versions only.

What have we learned through planning the Basel conference? First and foremost, Basel already has “green defaults”; for example, 100% of the electric power in Basel originates from renewable energy (hydro, solar, and wind). Basel is a nuclear-free city. But we also made progress in pushing ourselves and others involved in organizing the conference. However, “green” and “sustainable” are as multifactorial, interdisciplinary, and complex as the topics that will be discussed at the conference. There is no simple recipe or “truth.” Strategies for a green(er) conference need to become more evidence-based and cost-effective in order for green conferences to become the default. Although we are not there yet, what matters most is that we keep the vision of a green conference high on the agenda, on par with running a smooth and well-organized event for cutting-edge research.
